# Data use in social science and medical articles around the world

**DOI:** 10.1093/pnasnexus/pgaf196

**Published:** 2025-06-19

**Authors:** Brian Stacy, Lucas Kitzmüller, Xiaoyu Wang, Daniel Gerszon Mahler, Umar Serajuddin

**Affiliations:** World Bank, Development Data Group, 1818 H St NW, Washington, DC 20433, USA; European Bank for Reconstruction and Development (EBRD), Office of the Chief Economist, 5 Bank St, London E14 4BG, United Kingdom; World Bank, Development Data Group, 1818 H St NW, Washington, DC 20433, USA; World Bank, Development Data Group, 1818 H St NW, Washington, DC 20433, USA; World Bank, Development Data Group, 1818 H St NW, Washington, DC 20433, USA

**Keywords:** statistical systems, data use in academia, natural language processing, global development, empirical research

## Abstract

Data-driven research is key to producing evidence-based public policies, yet little is known about where data-driven research is lacking and how it can be expanded. We propose a method for tracking academic data use by country of subject in English-language social science and medicine articles, applying natural language processing to a large corpus of academic articles. The model’s predictions produce country estimates of the number of articles using data that are highly correlated with a human-coded approach, with a correlation of 0.99. Analyzing more than 140,000 academic articles, we find that high-income countries are the subject of ∼50% of all papers using data, despite only making up around 17% of the world’s population. Finally, we classify countries by whether they could most benefit from increasing their production or use of data, with the former applying to many poorer countries and the latter to many wealthier countries.

Significance Statement This study contributes to the literature on global patterns of data-driven research in five ways. First, it expands the analysis to a larger sample size of articles and disciplines than previous literature. Second, it employs natural language processing to identify articles using data, enabling an understanding of where empirical research is lacking. Third, it examines the relationship between data-driven research and GDP, population, and statistical capacity, addressing omitted variable bias by incorporating controls for nondata-driven research. Fourth, it classifies countries based on their potential to benefit from increasing data production or data use, providing a framework for prioritizing interventions. Fifth, it identifies specific measures countries can adopt to increase data-driven research, including improving statistical capacity and enhancing data accessibility.

## Introduction

In recent decades, the amount of data produced has grown rapidly, generating ample opportunities for evidence-based policymaking (1). While raw data hold value, their greatest impact emerges when analyzed into actionable insights that shape policies and accountability. Researchers play a pivotal role in this process, digesting data to generate new knowledge and influence policy. Data-driven research has demonstrably improved lives (2); for example, in Brazil, research insights increased the adoption of effective policies by municipal governments by 10 percentage points (3).

Though countries surely can learn from data-driven research conducted on other countries, localized evidence is often more useful (4) and there is a renewed interest in the need for localized policies (5). Without data-driven research on a country, there is a risk that policies will be less targeted towards the conditions of the country, and ultimately worse at improving outcomes. Yet very little is known about where there is missing data-driven evidence and how governments can best stimulate an evidence base for local decision-makers. This paper attempts to fill these gaps by addressing two questions: (i) which countries are the subject of English-language data-driven research papers in the fields of social science and medicine? and (ii) how can countries increase their national evidence base?

We focus on data-driven research due to the increasing importance of data for policy making and the specific policies that are needed to increase the production and use of data, such as boosting statistical capacity and improving data literacy. We constrain our analysis to English-language articles due to their global dominance and the unavailability of other languages in our data. We focus on social science and medicine articles because data in these fields are commonly produced by governments with the aim of guiding public policy. We discuss possible limitations and biases these constraints may cause on the results.

To answer the first question we pose, we introduce a new method for measuring data use in research articles using natural language processing (NLP). The approach makes use of 1 million English-language articles spanning 216 countries. Our model, trained on human-coded data, achieves 87% accuracy and correlates highly (0.99) with human classifications at the country level. Applying the model to around 140,000 academic articles, we find that data-driven research is heavily concentrated in high-income countries, which account for ∼50% of all articles despite comprising only 15% of the global population. Low-income countries, representing 10% of the population, account for just 5% of such articles.

For the second question, we identify statistical capacity as a key driver of data-driven research, even after controlling for GDP per capita, population, and other factors. Specific data sources—such as geospatial data and recent censuses and surveys—are associated with increased academic output. Countries can enhance their evidence base by producing critical datasets or improving data accessibility and literacy. To guide such efforts, we classify countries into four categories: deserts (low data use and production), swamps (high production but low use), oases (high use but low production), and lakes (high use and production), revealing distinct regional and income-based patterns.

Previous research has highlighted gaps between countries in economic research output and noted that richer countries are the subject of more economic research. For instance, Robinson et al. (6), Das et al. (7), and Porteous (8) examine which countries are studied most by economists using the EconLit database, finding that GDP per capita and population size are important determinants. Cameron et al. (9) and Sabet and Brown (10) extend this to note that impact evaluations are highly uneven across countries as well. Phillips and Greene (11) show conflict research is biased towards Western countries, while Courtioux et al. (12) show that academic research is highly related to public investments in scientific research. Tian (13) examined the use of statistics in national policy documents but used keyword matching rather than NLP to identify data use.

We contribute to the literature in five ways: (i) we scale up the sample size and look at several fields of interest rather than only economics; (ii) we use NLP to identify papers that use data, which is key to understanding where empirical research is lacking; (iii) we identify the relationship between papers using data and other variables, such as GDP, with less concern of omitted variable bias than prior studies by controlling for the number of articles *not* using data; (iv) we classify countries by whether they are likely to most benefit from expanding their data use or their data production; and (v) we indicate measures countries can take to increase data research.

## Results

### Data use around the world

**Fig. 1.**
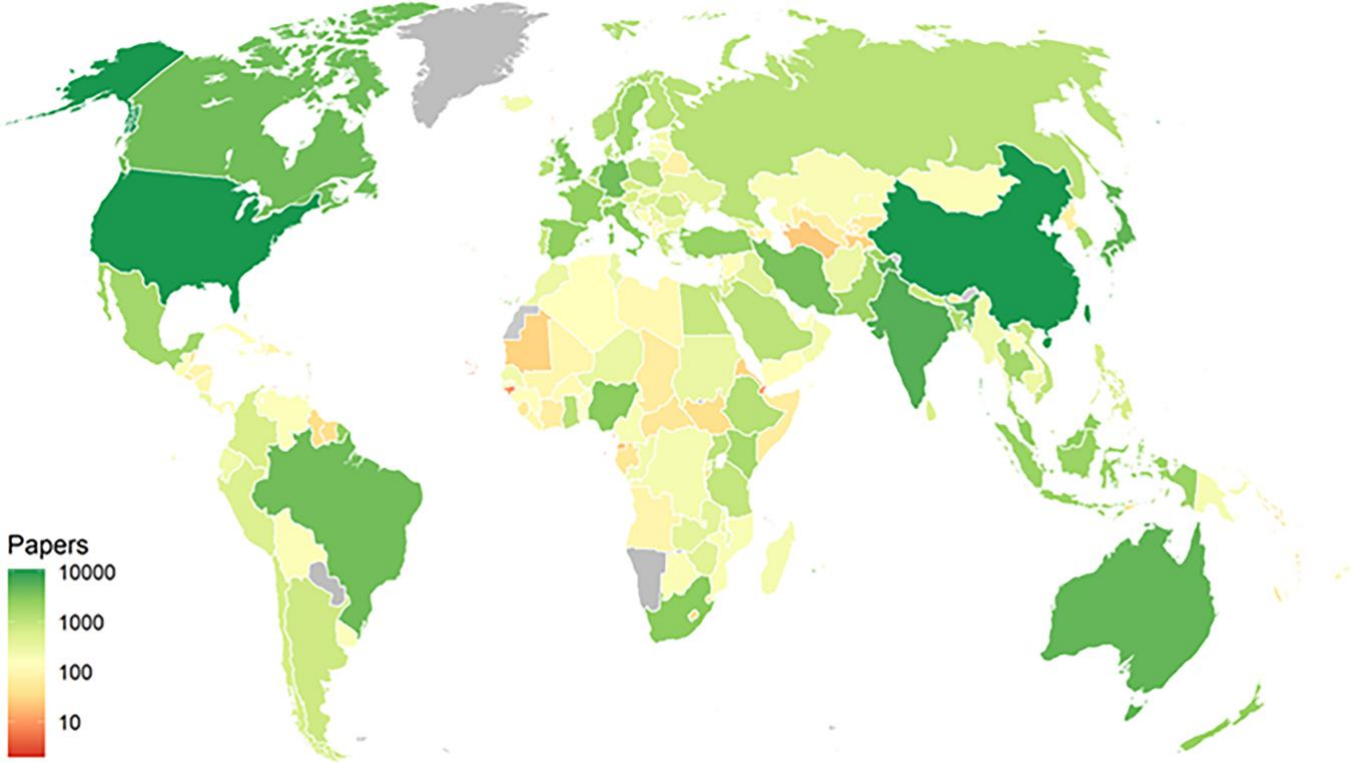
Number of English-language articles using data in social science or medicine, by country of subject (2000–2020); 140,085 articles included.

**Fig. 2.**
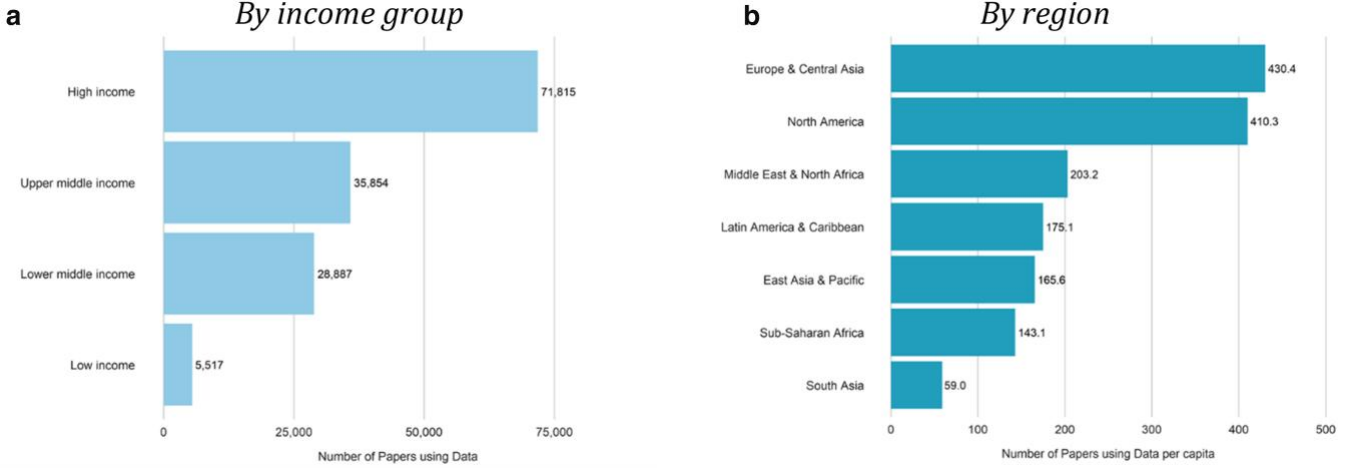
Number of articles using data by income group and region (2000–2020); 140,085 articles using data included. a) By income group and b) by region.

The distribution of data-driven English-language social science and medicine research articles (which we refer to in short as data-driven articles or articles using data) is highly uneven, with just five countries accounting for over 25% of all data-driven articles. The United States and China lead with more than 12,000 data-driven articles each, while India, Australia, and Japan round out the top five (Fig. 1). In contrast, the 50 least-researched countries collectively account for <1% of data-driven articles. High-income countries, comprising 17% of the global population, are the focus of ∼50% of data-driven articles, while low-income countries, representing 10% of the population, account for just 5%. Regionally, Europe and Central Asia have the highest per capita output, while South Asia has the lowest (Fig. 2).

### Relationships to development outcomes

**Fig. 3.**
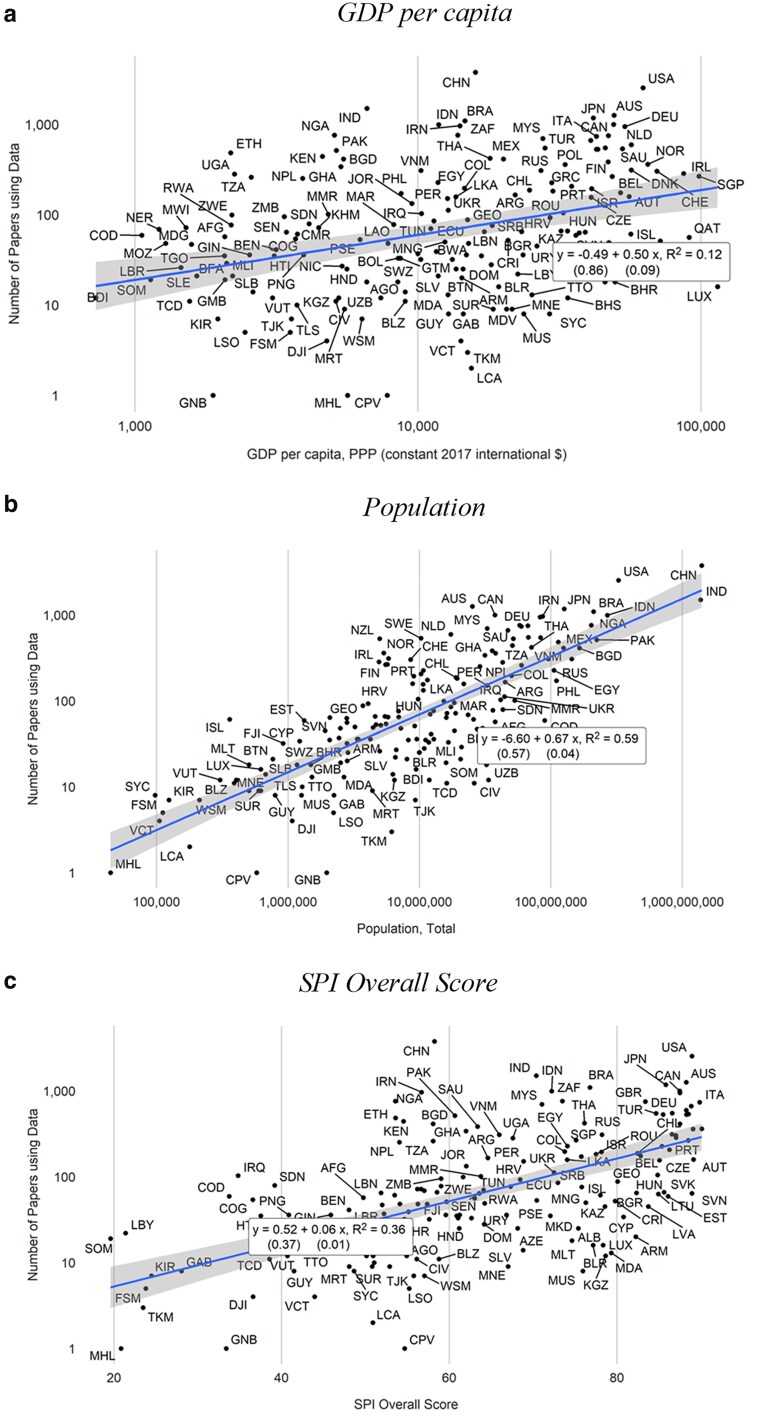
Relationship between articles using data and development outcomes. a) GDP per capita; b) population; (c) SPI overall score. Data on GDP, population, and SPI are retrieved from the World Bank’s World Development Indicators. Regressions include all papers using data from 2017 to 2019 (36,695 total articles). One hundred and sixty-eight countries are included.

Using around 37,000 articles published between 2017 and 2019, we formally test the predictors of data-driven research by regressing the number of English-language social science and medicine articles using data on likely factors. Figure 3 shows bivariate regressions of log number of data-driven articles on log GDP, log population, and the Statistical Performance Indicators (SPI) overall score—a measure of the performance of statistical systems for 174 countries ranging from 0 (worst) to 100 (best) (14, 15). The relationship with GDP per capita reveals an elasticity of 0.5, suggesting that a 10% increase in income corresponds to a 5% increase in the number of data-driven articles. Population is even more predictive, with an elasticity of 0.67, explaining 59% of the variation in data-driven research. The SPI score is also positively related to academic output. A 10-point increase in the SPI score, which is roughly equivalent to moving from the median score to the 65th percentile, corresponds to a 0.6% rise in the number of data-driven articles.

Table 1 displays the results from regressions of the data on data-driven articles on log GDP per capita, log population, and SPI scores. The regressions are done at the country level, with the log number of data-driven articles as the dependent variable. Column (1) includes only GDP per capita and population, with these two factors alone accounting for 75% of the variation in data-driven research output. The estimated elasticity of 0.58 for GDP per capita is quite similar to that estimated by Das et al. (7) (elasticity = 0.617) who examined economics articles written between 1985 and 2005, suggesting a relatively stable relationship between income and academic output. The estimates are larger than that found in Porteous (8) (elasticity = 0.361) found among only African countries.

**Table 1.** Relationships between empirical papers and development outcomes.

	(1) Log # of articles using data	(2) Log # of articles using data	(3) Stacked regression of articles using data (Poisson regression)	(4) Log # of articles using data	(5) Log # of articles using data	(6) Log # of articles using data
Log GDP per capita	0.58***	0.63***	0.60***	0.40***	0.18**	0.15*
	(0.05)	(0.05)	(0.06)	(0.07)	(0.07)	(0.07)
Log population	0.70***	0.66***	0.66***	0.40***	0.18**	0.15*
	(0.03)	(0.03)	(0.04)	(0.07)	(0.07)	(0.07)
Country official language is English		0.74***	0.34*	0.77***	0.48***	0.47***
		(0.13)	(0.15)	(0.12)	(0.11)	(0.11)
Log GDP per capita × top 500 articles			0.24*			
			(0.10)			
Log GDP per capita × top 200 articles			0.37+			
			(0.21)			
Log GDP per capita × top 100 articles			0.47*			
			(0.22)			
Log GDP per capita × citation count of articles using data			0.17+			
			(0.09)			
SPI overall score				0.02***	0.01+	
				(0.01)	(0.01)	
Log qualitative papers					0.55***	0.57***
					(0.08)	(0.08)
SPI data sources score						0.01**
						(0.00)
(Intercept)	−12.4***	−13.50***	−11.90***	−11.71***	−5.44***	−4.91***
	(0.8)	(0.79)	(0.89)	(0.89)	(1.21)	(1.22)
Obs.	166	166	830	166	166	166
Unit of analysis	Country Level	Country Level	Stacked Country Level	Country Level	Country Level	Country Level
*R* ^2^	0.751	0.784		0.811	0.865	0.865
*R* ^2^ adj.	0.748	0.780		0.806	0.861	0.861

All outcomes are logged. GDP, population, and SPI data are retrieved from the World Bank’s World Development Indicators. Data on English as an official language are sourced from the CIA Factbook. Regressions include all papers using data from 2017 to 2019 (36,695 total articles). Log GDP X Top 500 Articles is estimated by stacking the count of all articles using data, the count of articles in the top 500 journals based on impact factor. An interaction term is included with GDP per capita, population, and other controls. The interaction with top 200, top 100, and citation weighted counts of articles is done similarly. Because of very low counts of articles published in top 100 journals by impact factor, the Poisson regression of the count of articles is used instead of OLS regression of the log number of articles.

SEs are robust to heteroscedasticity. *** = 0.001 level; ** = 0.01 level; * = 0.05 level; + = 0.1 level.

Column (2) additionally controls for whether English is an official language of the country. Because our analysis is restricted to English-language articles, a concern could be that we undercount the number of data-driven articles in non-English speaking countries, which could be related to both population and GDP per capita, potentially biasing the relationship between data use and income or population. English as an official language is highly predictive of the number of articles using data. This reflects that a larger share of articles in countries where English is not an official language is written in other languages, but also that non-English speakers have a harder time writing in English and hence may produce less articles than native English speakers (16, 17). Estimates of the relationship between GDP per capita and population are very similar when including this additional control.

Column (3) examines whether the relationship between national income and research output varies with journal prestige. To do this, we restructure the data in a “stacked” format, where each country appears multiple times: once with the total count of data-driven articles and again with the count of articles published in top-tier journals—specifically, the top 500, top 200, and top 100 journals ranked by impact factor—as well as a citation-weighted count of articles. Dummy variables flag each of these stacked observations, and we interact these dummies with log GDP per capita to test whether the income elasticity differs across higher-quality publication measures. The interaction term for top 500 journals is positive and statistically significant (0.24), suggesting that GDP per capita has a stronger association with publication in higher-ranked journals than with overall data-driven research output. The magnitude of the interaction increases for top 200 and top 100 journals, indicating a steeper income gradient for more selective outlets. This suggests that researchers from higher-income countries are more likely to publish in prestigious journals and gain recognition.

Columns (4)–(6) examine the relationship between the performance of a country’s statistical system and the number of data-driven articles. Column (4) includes the SPI overall score as an additional control. The results show that better-performing national statistical systems are associated with greater academic output. Adding the SPI score explains about 3% of the previously unexplained variation in data use. Conditional on GDP per capita and population, a 10-point increase in SPI overall scores (on a 0–100 scale) is associated with a 0.2 log point increase in data-driven articles—approximately equivalent to a 20% absolute increase.

It is possible that omitted variables are behind this result. For example, it could be the case that countries that are of general interest to the research community also happen to have high statistical performance. We leverage that we have an estimate of the number of articles not using data—referred to as qualitative articles—to control for all factors that are likely to influence overall research output in a country unrelated to statistical performance. The number of qualitative articles is calculated as the number of total articles minus any articles using data, based on our NLP model. The fifth column of Table 1 additionally controls for the log number of qualitative articles. The SPI overall scores are still statistically significant at the 10% level, as is country population. In this model, a 1% increase in GDP per capita has the same impact as an 18-point increase in the SPI. Though this may sound small, it is arguably easier for countries to increase their SPI by 18 points (roughly the improvement seen by the average country between 2016 and 2023) than to grow their economy by 1% more. The former could be done by producing a few key surveys or making data more easily accessible. Column (6) examines the relationship with one of the pillars of the SPI, data sources, which covers the availability of recent censuses, surveys, academic data, and geospatial data. The data sources index from the SPI is also correlated with data use in academia, conditional on log GDP, population, and the number of qualitative papers. Similar regressions using the other pillars of the SPI, such as data services and data infrastructure, are not associated with the number of articles using data in a similar regression, suggesting that it is the availability of particular data sources that is helpful in boosting academic data use.

**Fig. 4.**
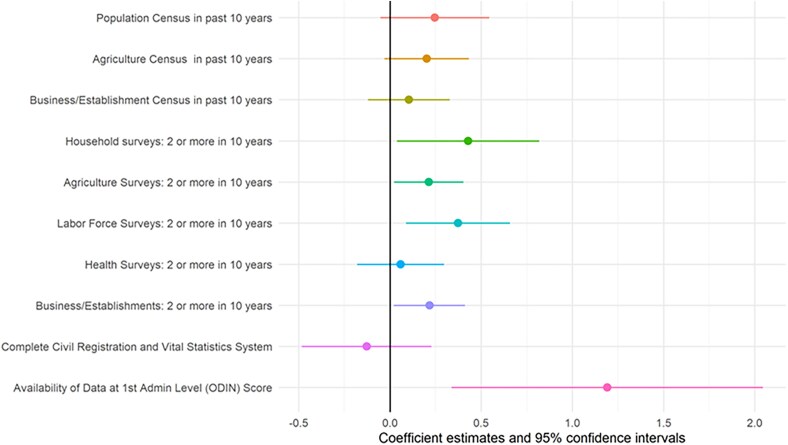
Relationship between number of papers using data and data sources. Results from cross-sectional regression controlling for log population, log GDP per capita, whether the country has English as an official language, and log number of qualitative papers. GDP and population data are retrieved from the World Bank’s Development Indicators, while the data source variables are from the SPI. The sample includes all papers using data between 2017 and 2019 (36,695 total articles). One hundred and sixty-eight countries are included. CIs are at the 95% significance level. Full regression results are presented in Table S2.

Using more detailed data from the SPI makes it possible to assess how the availability of specific data sources relates to academic output. Figure 4 shows the linear regression coefficient for the availability of each of the 10 data sources considered by the SPI, conditional on log GDP per capita, log population, and log of qualitative papers, and whether English is an official language of the country. The 10 data sources include: population census, agriculture census, business/establishment census, household consumption/income survey, agriculture survey, labor force survey, health survey, business/establishment survey, civil registration and vital statistics system (CRVS), and geospatial data at the Admin 1 (usually province/state) level.

The regression estimates indicate that the most important predictor of academic data use is the availability of data at the first administrative (Admin 1) level, which is associated with a 1.19 log-point increase in academic output using data. This suggests that a 10% increase in data availability at the Admin 1 level is associated with an ∼13% increase in academic output (since exp(0.119) ≈ 1.127). The availability of an agriculture census in the past 10 years is associated with a 0.20 log-point increase in data use, significant at the 10% level, implying a 22% increase in academic output (exp(0.20) ≈ 1.22). The availability of two or more household or labor force surveys in the past 10 years is associated with a 0.43 and 0.37 log-point increase in output, respectively—implying increases of roughly 54% (exp(0.43) ≈ 1.54) and 45% (exp(0.37) ≈ 1.45)—both significant at the 5% level. These effects likely underestimate the full potential impact of data investments, as some countries with relevant data sources may still face low uptake or visibility of their data products. We return to this issue in the following section.

### Data use vs. data production

The prior analysis suggested that countries can increase the number of data-driven academic articles they are subject to by producing relevant data sources. Yet some countries are already producing relevant data, and their primary obstacle preventing more data-driven research may be lack of use of existing data. It can be of interest for countries to know whether lack of data use or lack of data is the most pressing issue, as the policies needed to boost data production differ from those needed to boost data use. Boosting data production of the national statistical system requires more financing for national statistical offices, better technical capacity of its staff, and at times better statistical laws to ensure their independence from other government bodies (1). Policies to boost data use, on the contrary, involve financially cheap (but not necessarily politically cheap) wins, such as making data more accessible for researchers. It also involves increasing the data literacy of academics.

Assessing whether countries have most to gain by improving data production or data use is challenging because data production and use are positively correlated and not entirely separable. Demand for data occasionally leads to more data being produced, which increases the use of data, which in turn stimulates more demand for data, and hence sets about a positive feedback loop. This codependence makes it difficult to assess the causal effects of boosting data production vs. data use. Instead, we will simply look at whether countries are performing above or below the median of countries with respect to data production and use.

When assessing data production, we again turn to the availability of various data sources, such as recent population and business censuses, household and health surveys, CRVS, and subnational geospatial data (for details, see (15)). We measure data use as the residual from the regression of number of articles on the country using data on log population, log GDP per capita, and the average number of qualitative papers for the country. Controlling for the number of qualitative papers of the country once again serves as a proxy for general research interest and research environment unrelated to data. This control also implicitly takes out other factors that could create a positive correlation between data use and data production, such as a country’s governance. If, for example, a country’s governance is equally important for the number of qualitative articles and quantitative articles, then our measure of data use should not be driven by a country’s governance. We additionally control for whether English is an official language of the country to account for increased production of English-language articles using data in these countries.

**Fig. 5.**
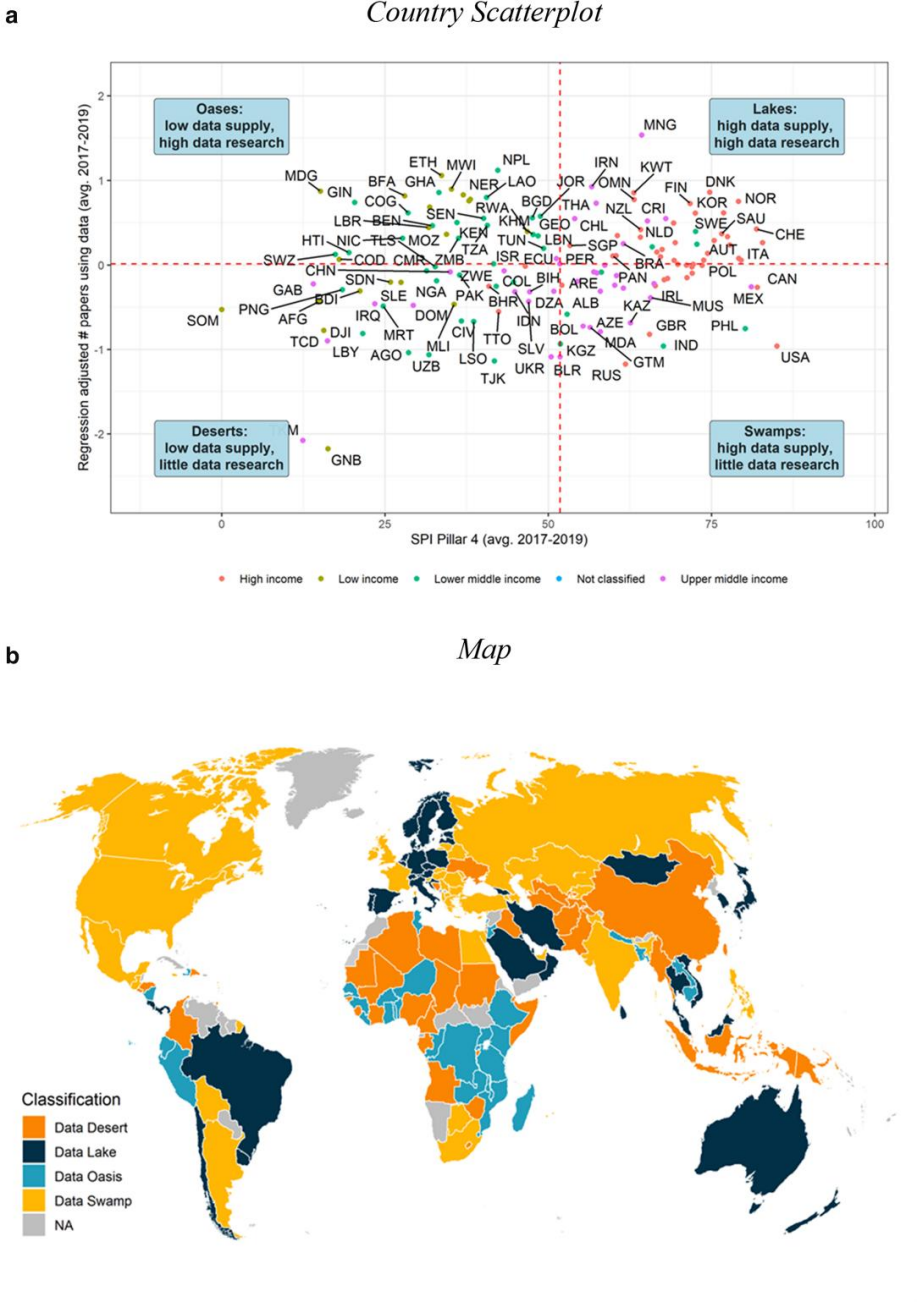
Relationship between data use and data supply. a) Country scatterplot and b) map. a) Plots on the vertical axis the residuals from a regression of the average number of data papers between 2017 and 2019 on log GDP, log population, whether English is an official language, and the log number of papers not using data (qualitative papers). The horizontal axis plots SPI Pillar 4, which tracks the availability of recent censuses, surveys, Civil Registration and Vital Statistics, and subnational data (for details, see (15)). Dashed lines represent median values. One hundred and forty-six countries with an SPI score and populations >1 million are included. Classifications based on 36,923 articles from 2017 to 2019. Data from the World Bank SPI.

Building on the semantics introduced by Porteous (8), who classified countries into research deserts and research oases, we classify countries into four groups: *Data deserts* have little data use and little data production, *data swamps* have high data production but little data use, *data oases* have high data use but little data production, and *data lakes* have high data use and high data production. Since we use median values in the two dimensions to create the categories, there are about as many countries belonging to each group (Fig. 5), yet there are large discrepancies by region and income group (Table 2). The poorest countries and poorest regions are more likely to be data deserts or data oases. These categories apply to more than 80% of low- and lower-middle-income countries, 92% of countries in sub-Saharan Africa, and only 6% of high-income countries.

**Table 2.** Data deserts, oases, lakes, and swamps by region and income group.

	Data deserts (%)	Data oases (%)	Data swamps (%)	Data lakes (%)	Number of countries
Region					
East Asia and Pacific	29	12	6	53	17
Europe and Central Asia	11	0	49	40	45
Latin America and Caribbean	26	21	26	26	19
Middle East and North Africa	35	18	12	35	17
North America	0	0	100	0	2
South Asia	33	33	17	17	6
Sub-Saharan Africa	38	54	8	0	39
Income group					
Low income	40	60	0	0	20
Lower-middle income	37	44	12	7	41
Upper-middle income	32	5	43	19	37
High income	6	0	32	62	47
Total	26	22	25	27	145

Share of countries within a region or income group that are classified as data deserts, oases, swamps, or lakes. One hundred and forty-five countries with an SPI score and populations >1 million are included. Classifications based on 36,923 articles from 2017 to 2019. For example, 29% of the 17 countries in East Asia and Pacific are classified as data deserts.

Low-income countries and countries in sub-Saharan Africa are around 50% more likely to be oases than deserts, and a large part of the world’s data oases are to be found in sub-Saharan Africa. This suggests that, despite data scarcity, a significant volume of data-driven articles is already produced on these countries, when factoring in their population size, income, and attention in qualitative scholarship. These countries may benefit most from bolstering their data production. This applies, for example, to Uganda, Ghana, Nepal, and Malawi. By contrast, nearly all upper-middle-income countries and countries in Latin America that have low data production are deserts rather than oases. That is, they face both data production and data use constraints. This concerns countries such as Libya, Turkmenistan, and El Salvador. Nearly all high-income countries are data lakes or data swamps, as are two-thirds of upper-middle-income countries. High-income countries are more likely to be lakes than swamps, while the opposite refers to upper-middle-income countries. Hence, the latter countries may have successfully improved data production but are lacking in data use, at least relative to the number of qualitative articles produced. This concerns countries such as the Russian Federation, the UK, France, Germany, and the United States. The Scandinavian countries are all classified as data lakes, meaning they have high production and high use of their data.

## Discussion

This paper suggests a method for measuring data use by researchers by classifying English-language academic articles using NLP. Our NLP model can classify whether social science and medicine articles use data with 87% accuracy and, if aggregated to the country level, match human-rated country-level classifications with a correlation of around 0.99. The obtained classification correlates strongly with alternative measures in the literature.

Our results can help countries better understand whether their statistical system is supporting the needs of researchers. Given that research is at the heart of evidence-based policy making, a statistical system oriented around making data available and easily accessible for these purposes is important. Countries could also use the results to learn from other countries and track progress or assess return on investment for funding related to statistical capacity building.

The strong relationship between income and journal prestige could have important policy implications, especially regarding the role of academic editors in shaping research priorities. Editors may incentivize research on higher-income countries, as shown by the link between GDP and publications in top-tier journals. Given their influence over what gets published, editors play a critical role in guiding the types of data that are used and prioritized in research. By encouraging submissions that include data from under-represented regions, editors could broaden the scope of academic discourse. This would not only promote geographic diversity but also help drive attention to underresearched areas, potentially reshaping global research agendas and incentives to collect data.

Our results face some limitations. First, the Semantic Scholar Open Research Corpus (S2ORC) database only contains English-language articles. Even if academic languages in other languages were accessible through other data sources, using them in this study would require human coders proficient in these other languages, increasing the cost and complexity of the study. The restriction to English-language articles matters for our count of the number of articles using data per country. While English is the dominant language of academic publications—one estimate holds that around 95% of social science papers written since 2000 are in English (18)—English publishing is less ubiquitous among nonnative English speakers. One study found that 90% of articles published by Colombian researchers are in English (17), while another found that nonanglophone researchers make on average 60% of their journal submissions in English (19).

Second, we constrained our analysis to social sciences and medicine. The current project is not aiming to make broad generalizations and identify patterns in *all* academic research, but it only aims to identify patterns in social sciences and medical research. Further studies would be needed to examine whether the same patterns hold in other disciplines. One study found that the share of studies on agriculture and immunology, both of which are excluded in our analysis, is greater in poorer countries. That said, given that poorer countries are also less frequently the subject of articles in physical science, the conclusion that richer countries are the subject of a lion’s share of articles using data would likely remain. Less than 4% of the world’s high-impact geoscience papers focus on Africa, there is less research on biodiversity in the world’s most biodiverse countries (which tend to be poorer), important countries for research in the tropics remain understudied (20, 21), and the contributions from poorer countries to experimental biology are low (22).

Third, because of computational limitations, only 1 million articles were analyzed out of a sampling frame of around 10 million articles. Future work could bring in these additional articles to improve precision. Fourth, while the NLP model was able to classify whether or not the articles made use of data with 87% accuracy, the model could be extended in ways to extract more information from the articles, including more details about the subject of study and which particular data sources may have been used (surveys, censuses, administrative data, geospatial information etc.).

## Materials and methods

### Data

Our primary data source is the S2ORC (23), which contains over 130 million English-language academic papers across multiple disciplines. These papers are collected from a variety of sources, including direct submissions from publishers, open archives such as arXiv and PubMed, and web crawls.

S2ORC offers several advantages as a data source, including its vast scale, broad disciplinary coverage, and rich metadata. Compared with other datasets such as PubMed Central, EconLit, or the ACL Anthology, S2ORC is not only significantly larger but also more diverse, spanning numerous academic fields rather than being restricted to specific domains (e.g. biomedical research, economics, or computational linguistics). However, a key limitation is that S2ORC includes only English-language papers, potentially excluding valuable research published in other languages. This restriction could result in underrepresentation of insights from non-English-speaking regions, which may affect the comprehensiveness of the analysis.

We place some restrictions on the articles to make them usable and relevant for our purposes. First, only articles with an abstract and parsed PDF or latex file are included in the analysis. The parsed PDF and latex file are important for extracting information such as the date of publication and field of study. This restriction eliminated many articles in the original corpus. Around 30 million articles remain after keeping only articles with a parsable (i.e. suitable for digital processing) PDF, and around 26% of those 30 million are eliminated when removing articles without an abstract. Second, only articles from the years 2000 to 2020 were considered. This restriction eliminated an additional 9% of the remaining articles. Finally, articles from the following fields of study were excluded, as we aim to focus on fields that are likely to use data produced by countries’ national statistical system: Biology, Chemistry, Engineering, Physics, Materials Science, Environmental Science, Geology, History, Philosophy, Math, Computer Science, and Art. Fields that *are* included are: Economics, Political Science, Business, Sociology, Medicine, and Psychology. This third restriction eliminated around 34% of the remaining articles.

From an initial corpus of 136 million articles, this resulted in a final corpus of around 10 million articles. Due to the intensive computer resources required, a set of 1,037,748 articles were randomly selected from the 10 million articles in our restricted corpus as a convenience sample. Summary statistics of the final sample of 1 million articles are available in Table 3.

**Table 3.** Summary statistics of article corpus (2000–2020): 1,037,748 articles included.

Field	Published in journal (1 = yes)	Data use (1 = yes)	Country identified (1 = yes)	Articles	Share of articles
Business	0.56	0.64	0.30	28,571	2.8
Economics	0.79	0.68	0.28	62,241	6.0
Medicine	0.96	0.85	0.10	840,920	81.0
Political Science	0.42	0.33	0.34	26,185	2.5
Psychology	0.75	0.70	0.14	44,191	4.3
Sociology	0.90	0.33	0.25	35,640	3.4

### Method

The empirical approach employed in this project utilizes text mining with NLP. The goal of NLP is to extract structured information from raw, unstructured text. We use NLP to extract the country of study and whether the paper makes use of data. We will discuss each of these in turn.

To determine the country or countries of study in each academic article, we employ two approaches based on information found in the title, abstract, or topic fields. The first approach uses regular expression searches based on the presence of ISO3166 country names. This approach is transparent, widely used in social science research, and easily extended to other languages. However, there is a potential for exclusion errors if a country’s name is spelled nonstandardly.

The second approach is based on Named Entity Recognition (NER), which uses machine learning to identify objects from text, utilizing the spaCy Python library. The NER algorithm is used in this project to identify countries of study in the academic articles. SpaCy supports multiple languages and has been trained on multiple spellings of countries, overcoming some of the limitations of the regular expression approach. If a country is identified by either the regular expression search or NER, it is linked to the article. Note that one article can be linked to more than one country.

The second task is to classify whether the paper uses data. We employ a supervised machine learning approach, where 3,500 publications were first randomly selected and manually labeled by human raters using the Mechanical Turk service (24).^a^ To make sure the human raters had a similar and appropriate definition of data in mind, they were given a set of instructions before seeing their first paper.

An image of the screen facing the MTurk workers and the printed instructions when classifying an article is presented in Fig. S1. The median amount of time that a worker spent on an article, measured as the time between when the article was accepted to be classified by the worker and when the classification was submitted, was 25.4 min. If human raters were exclusively used rather than machine learning tools, then the corpus of 1,037,748 articles examined in this study would take around 50 years of human work time to review at a cost of $3,113,244, which assumes a cost of $3 per article as was paid to MTurk workers.

Next, we train a model on the 3,500 labeled articles. We use a distilled version of the Bidirectional Encoder Representations for Transformers (BERT) model to encode raw text into a numeric format suitable for predictions (25, 26). BERT is pretrained on a large corpus comprising the Toronto Book Corpus and Wikipedia. Of the 3,500 articles that were hand coded by the MTurk workers, we feed 900 to the machine learning model. The model only uses the abstract, title, and topic fields from the papers and is therefore not constrained to open-access papers. Nine hundred articles were selected because of computational limitations in training the NLP model. We classify an article as “uses data” if the model predicted an article used data with at least 90% confidence.

We compare the performance of the models to the classification by the human raters. We consider the human raters as giving us the ground truth. This may underestimate the model performance if the workers at times got the allocation wrong in a way that would not apply to the model. For instance, a human rater could mistake the Republic of Korea for the Democratic People’s Republic of Korea. If both humans and the model perform the same kind of errors, then the performance reported here will be overestimated.

**Fig. 6.**
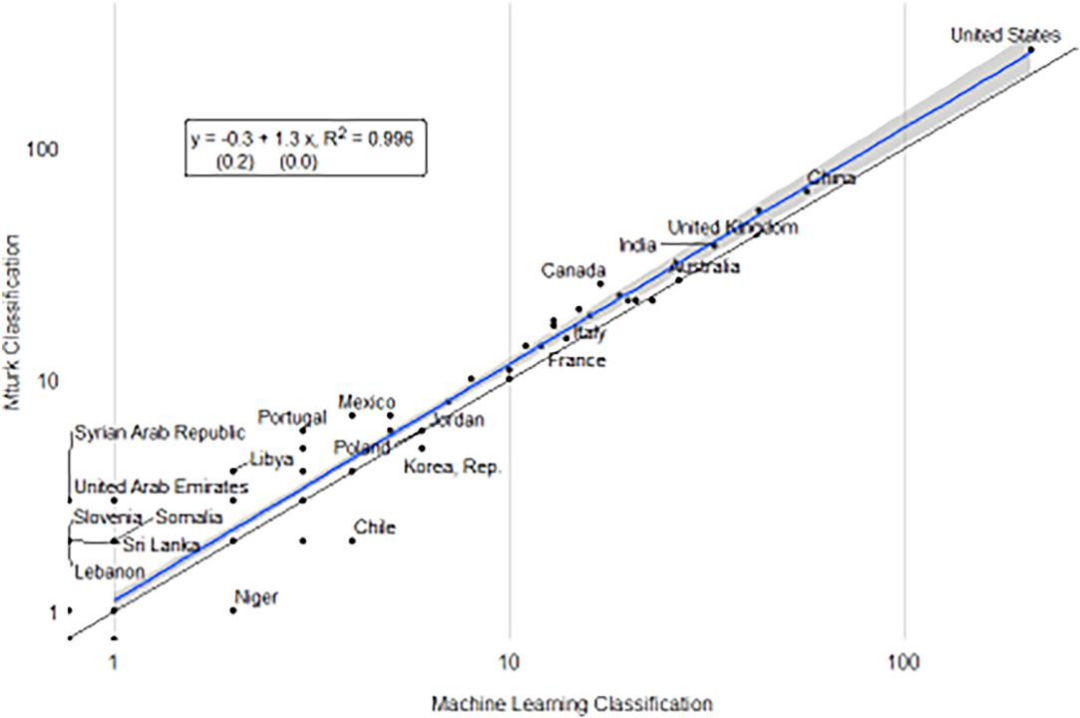
Comparison of human classifications of data use to NLP predictions. The horizontal axis shows the number of articles on a country using data as predicted by the NLP model. The vertical axis shows the number of articles on a country using data as classified by the human raters (Mturk workers).

The model was able to predict whether an article made use of data with 87% accuracy evaluated on the set of articles held out of the model training. The correlation between the number of articles written about each country using data estimated under the two approaches is given in Fig. 6. The number of articles represents an aggregate total of research output using data for each country summed from the corpus of papers that were not used to train the model. The Pearson correlation between the human raters and the NLP predictions is 0.996.

To make the performance of the model more concrete, we consider the output returned for three example articles. These articles were not in the training set of articles, so the model has not previously seen them. The first article, Perlman (27), titled, “The Legal Ethics of Metadata Mining” is a law essay examining the ethics of metadata mining. This article has data (or metadata) as the subject of the article, but does not actually use data to perform analysis, making it potentially difficult to machine classify. In the authors’ judgment, this article should not be classified as using data. The NLP model also evaluated that this article did not use data.

**Fig. 7.**
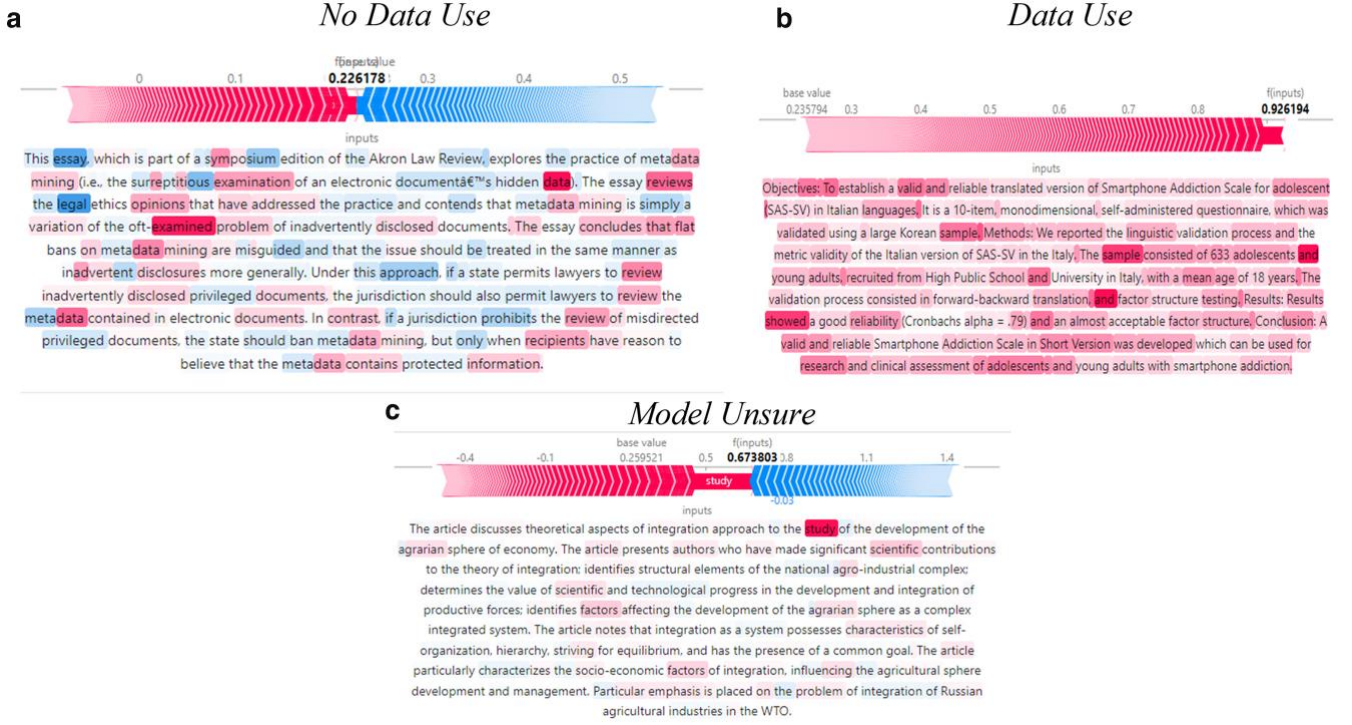
Article examples. The left panel is a sample from Perlman, Andrew M. “The Legal Ethics of Metadata Mining.” Akron Law Review 43 (2009). The right panel is a sample from De Pasquale, Concetta, Federica Sciacca, and Zira Hichy. “Italian validation of smartphone addiction scale short version for adolescents and young adults (SAS-SV).” Psychology 8, no. 10 (2017). The bottom panel is a sample from Smirnov, Anatoly A., and Irina V. Stukova. “Determinants of integration approach in the agrarian sphere development in contexts of transformation.” *Review of European studies* 7, no. 8 (2015) (28): 8. a) No data use; (b) data use; and c) model unsure.

Figure 7 shows words that the model viewed as indicating data use (in red) and likely indicating an article does not use data (in blue) using the SHAp (SHapley Additive exPlanations) package in python (Lundberg and Lee [28]). While the model picked up keywords like data and examined (highlighted in red), indicating data use, the NLP algorithm also picked up other key words such as legal and review, which had a reduced likelihood of using data. On balance the predictions of the NLP model indicated the article did not use data. Using an alternative approach, such as flagging key words in the article abstract like data, would have flagged this article as using data and given the incorrect classification.

The second article, De Pasquale et al. [29], titled, “Italian validation of smartphone addiction scale short version for adolescents and young adults (SAS-SV),” was classified as using data. In the authors’ judgment, this was a correct classification. The article picked up on key words such as sample, showed, scale, and numeric values, indicating data use (highlighted in red). The third article, Smirnov an Stukova [30], titled, “Determinants of integration approach in the agrarian sphere development in contexts of transformation” was given a probability of using data of 0.67, which was below the threshold of 0.9 used to assign a label of data use. However, the model thought the article was more likely than not to use data. In fact, the article does not use data, indicating that the model can be ambiguous about the status of an article.

Applying the NLP model to 140,000 articles identified with a country, we compare our results to prior estimates. Das et al. (7), using 76,000 empirical economics papers (1985–2005) from EconLit, has a correlation of 0.62 with our approach, despite differences in coverage and subjects. Similarly, our country rankings correlate at 0.87 with those from Porteous (8), who analyzed economics articles from 54 African countries (2000–2019). Compared with the National Science Foundation’s (31) 2018 estimates of scientific and technical articles, our NLP model achieves a correlation of 0.9.

### Hypotheses

Building on a model introduced in Porteous (8), we examine the relationship between the total amount of academic research using data and other development outcomes. In a simple framework, academics produce research using data, with the benefits of that research scaling with the size of a country’s population and GDP. This relationship can be motivated by the idea that researchers may aim to produce work that impacts the largest possible number of individuals or that the prestige of academic output grows with the size of the population or income level of a country it pertains to. Additionally, as Porteous (8) discusses, larger and more complex economies provide more topics to study, as their size and diversity increase the range of sectors and policy environments available for analysis. However, academic research exhibits diminishing returns, as the value of additional research decreases when there is already a large body of existing work.

The marginal cost of conducting research using data varies across countries and depends on several factors, such as the stock of existing data available for research, the cost of data collection, and differences in the cost of fieldwork. Academics are assumed to allocate their efforts across countries such that the net benefit of additional research is equalized across locations. This framework motivates a reduced-form model in which the total amount of research on a country is related to its population, economic size, and factors affecting the cost of conducting research. Specifically, we estimate the following regression:


(1) $$\log\;(R_{i})=\alpha +\;\beta_{1}\log(N_{i})+\;\beta_{2}\log(Y_{i})-\beta_{3}X_{i}+\;\epsilon_{i}$$

Equation (1) can be estimated using OLS, and the coefficients *β*_1_, *β*_2_, *and β*_3_ can be interpreted as the elasticities of population size, economic output, and factors affecting costs of gathering data with respect to academic output using data. As a measure of the cost of gathering data for research, we use as a proxy variable the World Bank’s SPI, a measure of the performance of statistical systems for 174 countries (14, 15). The SPI overall score is based on five pillars: Data Use, Data Services, Data Products, Data Sources, and Data Infrastructure. Data Use measures the availability of key information for international reporting. Data Services assess the availability of online platforms for data access. Data Products focus on the availability of SDG indicators. Data Sources examine the availability of various sources, such as censuses, surveys, and administrative data, excluding data collected directly by researchers. Data Infrastructure includes whether the statistical system is following internationally recognized standards and methodologies. These pillars are supported by 22 dimensions and 51 indicators, providing a comprehensive measure of a country’s statistical capabilities. Scores on the SPI are on a scale of 0–100, where countries scoring near 100 are the best performing systems and countries scoring closer to zero are the lowest performing systems. Because the term *β*_3_ for *X_i_* in equation (3) is structured as the elasticity with respect to costs of data collection, and because SPI scores are formulated so that better performing systems score higher, the coefficient on the SPI score in a regression will have the opposite sign as *β*_3_.

We estimate equation (3) using a dataset containing 167 countries with data available on all key variables. The number of articles is the 3-year average count of all articles produced using data for each country between 2017 and 2019, representing around 37,000 articles over that period and identifiable with a country. We use a 3-year average rather than a single-year value for 2019, because the production of articles can be volatile for a single-year observation. Population, GDP per capita, PPP, and SPI scores are all for the year 2019. Whether the country has English as an official language is included as a control as well. There is a strong relationship between the number of articles produced using data and GDP per capita, population, and the overall SPI score of a country.

## Sensitivity to methodological choices

### Panel analysis

Our analysis may face omitted variable bias if countries with high statistical capacity share traits unrelated to qualitative articles. To address this, we use country fixed effects and year dummies in OLS regressions, focusing on within-country variation over time. While this approach reduces bias, it may exclude relevant variation from consistently high-output countries.

Under this specification, the elasticity between academic output using data and log GDP per capita remains 0.4–0.6, while population is generally not significant (Table S3). SPI scores, available only from 2016, are also not significant due to the short time frame. To extend the analysis, we incorporate older statistical capacity indicator data into an “extended SPI” index, revealing a 10-point increase in the extended SPI score is linked to a 0.1% rise in data-driven academic output. This result, significant at the 5% level, aligns with cross-sectional findings, as does the extended SPI data sources score.^b^

### Excluding medical articles

Medical articles make up around 81% of all articles in our corpus (though this number falls to 63% when considering articles with a country identified). A concern could be that medical articles receive too much weight in our calculations of country scores. As a check, we compare our main results when dropping all medical articles. Except for countries with very few articles classified as using data, there is a very high correlation between the measures based on all fields and measures excluding medicine (Fig. S2). This makes us comfortable that the high share of medical articles is not driving our results.

Figure S3 shows the relationships between the number of articles per country using data for all subjects and for each subject, respectively, between 2000 and 2020. In all cases, academic output using data is strongly correlated across subjects. The correlation between the number of medical articles using data and economics is around 0.94. The subjects with the greatest correlation in articles using data are Political Science and Sociology with a correlation close to 0.97. Economics and Psychology have the lowest correlation (0.74).

## Supplementary Material

pgaf196_Supplementary_Data

## Data Availability

The datasets analyzed in the current study are available in the World Bank Data Catalogue. https://datacatalog.worldbank.org/search/dataset/0065200/Data-Use-in-Academia-Dataset. The code to reproduce all tables and figures is available in the World Bank Reproducible Research Repository.

## Notes

a A stratified sample of articles for the training set was taken. Of the articles, 15% were medical, 15% psychology, 25% economics, 25% political science, 10% business, and 10% sociology articles.

b The SCI includes fewer indicators than the SPI (25 vs. 51), with 11 overlapping (e.g. national accounts base year, CPI, population census, and CRVS availability). Using these, the extended SPI series covers 35 indicators back to 2004, spanning data use, services, products, sources, and infrastructure. The extended SPI correlates with the official SPI at ∼0.90.

## Preprints

This manuscript was posted on a preprint: http://documents.worldbank.org/curated/en/099503001172493286/IDU1ad0286b61aec014.
