# Supplementary material for: Data use in social science and medical articles around the world
Source: PNAS Nexus. 2025 Jun 19;4(6):pgaf196. doi: 10.1093/pnasnexus/pgaf196 (PMC12198491; doi:10.1093/pnasnexus/pgaf196)
Supplement: pgaf196_Supplementary_Data [file pgaf196_supplementary_data.docx]

Supplementary Materials for

**Data Use in Social Science & Medical Articles around the World**

Brian Stacy* *et al.*

*Corresponding author. Email: [bstacy@worldbank.org](mailto:bstacy@worldbank.org)

Supplementary Figs. S1 to S5, Tables S1 to S3

Fig. S1.

Amazon Mturk Prompt
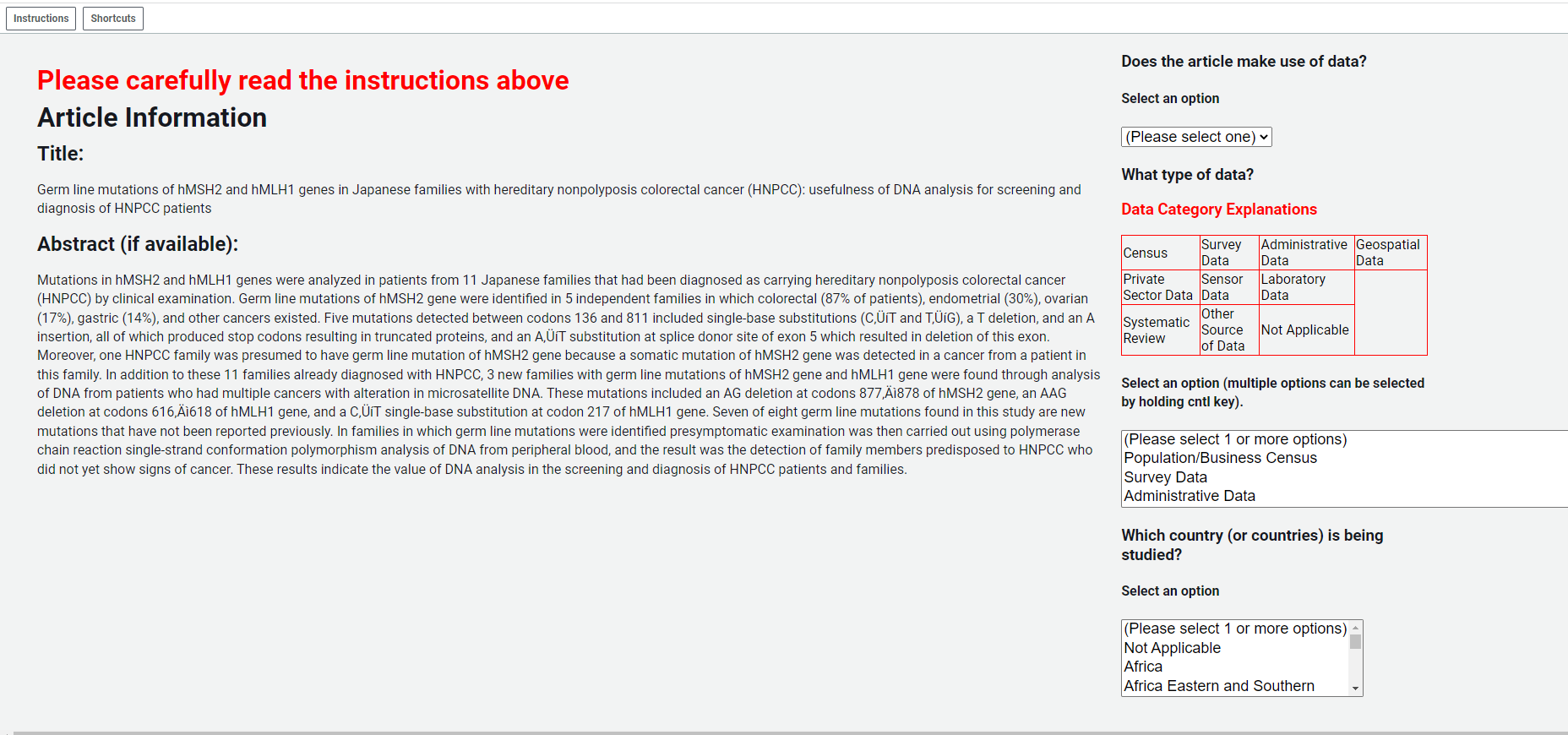


*Each of these documents is an academic article. The goal of this study is to measure whether a specific academic article is using data and from which country the data came.*

*There are two classification tasks in this exercise:*

1. *Identifying whether an academic article is using data from any country*
2. *Identifying from which country that data came.*

*For task 1, we are looking specifically at the use of data. Data is any information that has been collected, observed, generated or created to produce research findings. As an example, a study that reports findings or analysis using a survey data, uses data. Some clues to indicate that a study does use data includes whether a survey or census is described, a statistical model estimated, or a table or means or summary statistics is reported.*

*After an article is classified as using data, please note the type of data used. The options are population or business census, survey data, administrative data, geospatial data, private sector data, and other data. If no data is used, then mark "Not applicable". In cases where multiple data types are used, please click multiple options.*

*For task 2, we are looking at the country or countries that are studied in the article. In some cases, no country may be applicable. For instance, if the research is theoretical and has no specific country application. In some cases, the research article may involve multiple countries. In these cases, select all countries that are discussed in the paper*.

*We expect between 10 and 35 percent of all articles to use data.*

Fig. S2.

Comparison Between All Papers Using Data and Non-Medical Papers Using Data


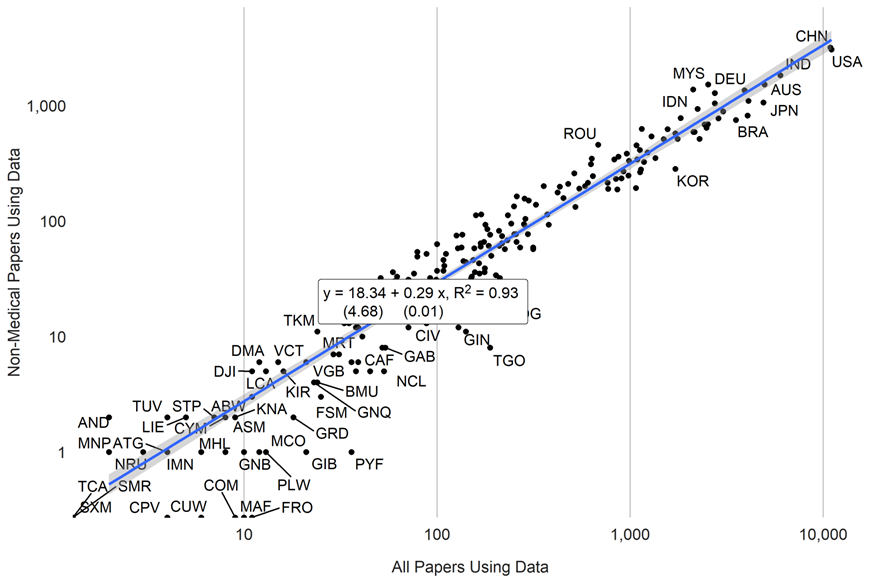


Fig. S3.

Correlation in Papers Using Data Across Subjects. 2000-2020.


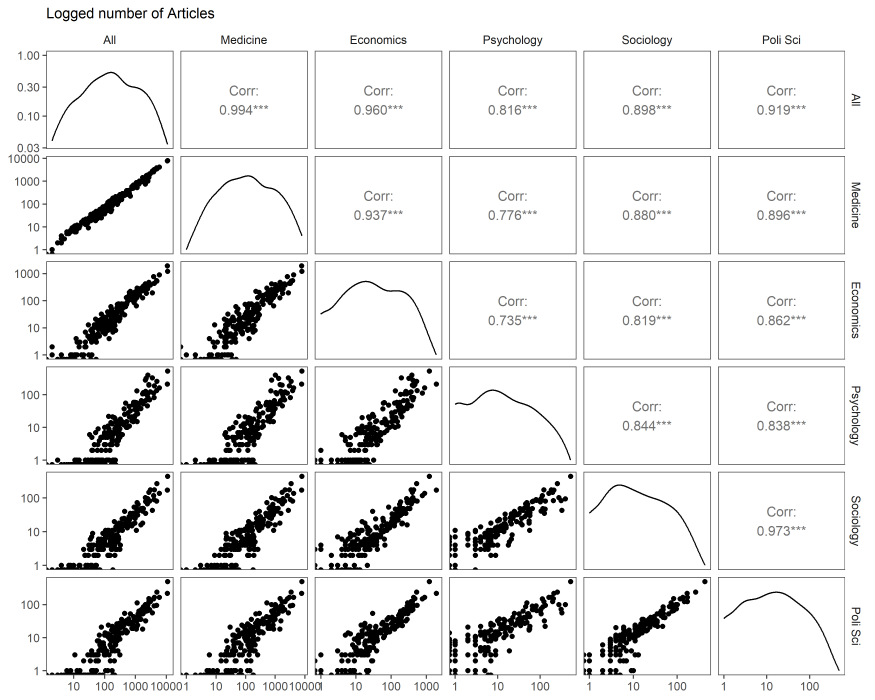


Table S1.

Country Production of Articles Using Data. Per capita and Total. 2017-19. 158 countries with populations greater than 1 million are included. Classifications based on 11,081 articles from 2019.

| Country | Articles using data per million persons | Total Number of Articles using data |
| --- | --- | --- |
| New Zealand | 35.55 | 133 |
| Norway | 22.56 | 98 |
| Ireland | 19.19 | 68 |
| Denmark | 17.89 | 89 |
| Sweden | 17.35 | 129 |
| Australia | 16.55 | 337 |
| Finland | 16.00 | 68 |
| Singapore | 15.55 | 86 |
| Estonia | 14.82 | 17 |
| Switzerland | 11.78 | 88 |
| Netherlands | 11.45 | 183 |
| Hong Kong SAR, China | 10.52 | 78 |
| Slovenia | 10.22 | 19 |
| Cyprus | 9.22 | 8 |
| Canada | 8.83 | 289 |
| Georgia | 7.88 | 27 |
| Latvia | 7.84 | 12 |
| Croatia | 7.63 | 26 |
| Lithuania | 7.52 | 15 |
| Israel | 7.14 | 52 |
| Malaysia | 7.10 | 200 |
| Greece | 7.03 | 67 |
| Portugal | 6.68 | 60 |
| Qatar | 6.65 | 20 |
| Botswana | 6.40 | 14 |
| Puerto Rico | 6.16 | 19 |
| Austria | 5.97 | 43 |
| Kosovo | 5.96 | 9 |
| North Macedonia | 5.62 | 7 |
| Mongolia | 5.16 | 14 |
| Eswatini | 5.13 | 4 |
| Belgium | 5.08 | 60 |
| Czechia | 4.87 | 43 |
| Oman | 4.78 | 17 |
| Spain | 4.69 | 211 |
| South Africa | 4.35 | 214 |
| Jordan | 4.14 | 49 |
| Italy | 4.10 | 238 |
| Slovak Republic | 4.03 | 10 |
| Bahrain | 4.02 | 4 |
| Germany | 3.80 | 273 |
| Jamaica | 3.79 | 5 |
| West Bank and Gaza | 3.77 | 18 |
| United Kingdom | 3.75 | 219 |
| Lebanon | 3.75 | 21 |
| Iran, Islamic Rep. | 3.71 | 266 |
| Kuwait | 3.68 | 12 |
| Serbia | 3.65 | 18 |
| Ghana | 3.60 | 110 |
| Hungary | 3.58 | 30 |
| Bosnia and Herzegovina | 3.57 | 10 |
| Saudi Arabia | 3.56 | 133 |
| Uruguay | 3.50 | 12 |
| Costa Rica | 3.41 | 16 |
| Korea, Rep. | 3.41 | 169 |
| Chile | 3.27 | 55 |
| Congo, Rep. | 3.23 | 15 |
| Romania | 3.15 | 47 |
| Poland | 3.14 | 134 |
| Bulgaria | 3.11 | 21 |
| Japan | 3.10 | 341 |
| Albania | 2.92 | 6 |
| Nepal | 2.91 | 66 |
| Kenya | 2.89 | 132 |
| Trinidad and Tobago | 2.85 | 2 |
| Panama | 2.84 | 10 |
| France | 2.72 | 165 |
| Timor-Leste | 2.60 | 3 |
| United States | 2.58 | 724 |
| Gambia, The | 2.52 | 4 |
| Sri Lanka | 2.42 | 42 |
| Armenia | 2.36 | 1 |
| Lao PDR | 2.22 | 19 |
| Turkiye | 2.18 | 157 |
| Uganda | 2.18 | 86 |
| Zimbabwe | 2.15 | 29 |
| Mauritius | 2.11 | 3 |
| Rwanda | 2.00 | 28 |
| Thailand | 1.96 | 134 |
| Tunisia | 1.94 | 21 |
| United Arab Emirates | 1.85 | 20 |
| Liberia | 1.74 | 9 |
| Brazil | 1.72 | 323 |
| Zambia | 1.72 | 33 |
| Ecuador | 1.63 | 19 |
| Moldova | 1.63 | 4 |
| Peru | 1.53 | 45 |
| Cambodia | 1.48 | 23 |
| Tanzania | 1.45 | 76 |
| Togo | 1.42 | 9 |
| Ethiopia | 1.41 | 177 |
| Nicaragua | 1.35 | 10 |
| Senegal | 1.33 | 22 |
| Colombia | 1.30 | 70 |
| Malawi | 1.27 | 21 |
| Papua New Guinea | 1.26 | 15 |
| Djibouti | 1.24 | 1 |
| Nigeria | 1.24 | 232 |
| Argentina | 1.23 | 57 |
| Indonesia | 1.23 | 339 |
| Gabon | 1.19 | 4 |
| Eritrea | 1.14 | 5 |
| Cuba | 1.12 | 12 |
| Libya | 1.12 | 2 |
| Benin | 1.11 | 11 |
| Mexico | 1.10 | 124 |
| Equatorial Guinea | 1.07 | 3 |
| Viet Nam | 1.07 | 101 |
| Haiti | 1.05 | 11 |
| Central African Republic | 1.02 | 3 |
| Niger | 0.98 | 26 |
| Bolivia | 0.93 | 12 |
| Guinea | 0.93 | 11 |
| China | 0.89 | 1,191 |
| Sierra Leone | 0.87 | 6 |
| Dominican Republic | 0.86 | 9 |
| Honduras | 0.84 | 10 |
| Ukraine | 0.84 | 33 |
| Azerbaijan | 0.83 | 9 |
| Bangladesh | 0.83 | 116 |
| Iraq | 0.83 | 32 |
| Kazakhstan | 0.81 | 13 |
| Cameroon | 0.79 | 24 |
| Pakistan | 0.77 | 177 |
| Lesotho | 0.75 | 2 |
| El Salvador | 0.74 | 4 |
| Egypt, Arab Rep. | 0.72 | 83 |
| Morocco | 0.72 | 27 |
| Russian Federation | 0.71 | 81 |
| Mauritania | 0.68 | 3 |
| Guatemala | 0.66 | 7 |
| Syrian Arab Republic | 0.66 | 16 |
| Myanmar | 0.63 | 40 |
| Kyrgyz Republic | 0.62 | 4 |
| Sudan | 0.61 | 26 |
| Belarus | 0.57 | 4 |
| Madagascar | 0.57 | 14 |
| Mozambique | 0.53 | 14 |
| Philippines | 0.52 | 52 |
| Afghanistan | 0.50 | 15 |
| Burkina Faso | 0.46 | 8 |
| South Sudan | 0.41 | 3 |
| Yemen, Rep. | 0.41 | 17 |
| Somalia | 0.40 | 6 |
| India | 0.36 | 466 |
| Venezuela, RB | 0.35 | 6 |
| Burundi | 0.34 | 4 |
| Mali | 0.34 | 8 |
| Algeria | 0.30 | 11 |
| Tajikistan | 0.25 | 2 |
| Chad | 0.23 | 3 |
| Congo, Dem. Rep. | 0.22 | 15 |
| Angola | 0.19 | 4 |
| Guinea-Bissau | 0.17 | 0 |
| Turkmenistan | 0.16 | 0 |
| Côte d'Ivoire | 0.14 | 3 |
| Korea, Dem. People's Rep. | 0.14 | 0 |
| Uzbekistan | 0.12 | 1 |

Note: Countries are divided into five quintiles. Countries shaded in dark orange have the lowest numbers of data use articles, countries in dark green have the highest. Papers include all papers using data years 2017-2019. Population data comes from the World Bank. Only countries with a total population of more than 1 million persons shown.

Table S2.

Relationship between Data Use in Academia and Data Sources from SPI.

|  | (1) | (2) | (3) | (4) | (5) | (6) | (7) | (8) | (9) | (10) |
| --- | --- | --- | --- | --- | --- | --- | --- | --- | --- | --- |
| Population Census in past 10 years | 0.25 |  |  |  |  |  |  |  |  |  |
|  | (0.15) |  |  |  |  |  |  |  |  |  |
| Agriculture Census in past 10 years |  | 0.20+ |  |  |  |  |  |  |  |  |
|  |  | (0.12) |  |  |  |  |  |  |  |  |
| Business/Establishment Census in past 10 years |  |  | 0.10 |  |  |  |  |  |  |  |
|  |  |  | (0.11) |  |  |  |  |  |  |  |
| Household surveys: 2 or more in 10 years |  |  |  | 0.43* |  |  |  |  |  |  |
|  |  |  |  | (0.20) |  |  |  |  |  |  |
| Agriculture Surveys: 2 or more in 10 years |  |  |  |  | 0.21* |  |  |  |  |  |
|  |  |  |  |  | (0.10) |  |  |  |  |  |
| Labor Force Surveys: 2 or more in 10 years |  |  |  |  |  | 0.37* |  |  |  |  |
|  |  |  |  |  |  | (0.14) |  |  |  |  |
| Health Surveys: 2 or more in 10 years |  |  |  |  |  |  | 0.06 |  |  |  |
|  |  |  |  |  |  |  | (0.12) |  |  |  |
| Business/Establishments: 2 or more in 10 years |  |  |  |  |  |  |  | 0.22* |  |  |
|  |  |  |  |  |  |  |  | (0.10) |  |  |
| Complete Civil Registration and Vital Statistics System |  |  |  |  |  |  |  |  | -0.13 |  |
|  |  |  |  |  |  |  |  |  | (0.18) |  |
| Availability of Data at 1st Admin Level (ODIN) Score |  |  |  |  |  |  |  |  |  | 1.19** |
|  |  |  |  |  |  |  |  |  |  | (0.44) |
| Intercept | -5.74*** | -5.60*** | -5.51*** | -6.32*** | -5.74*** | -5.30*** | -5.64*** | -5.48*** | -5.89*** | -5.52*** |
|  | (1.29) | (1.30) | (1.32) | (1.35) | (1.28) | (1.26) | (1.31) | (1.29) | (1.32) | (1.26) |
| Log GDP per capita | 0.25*** | 0.25*** | 0.27*** | 0.30*** | 0.27*** | 0.23*** | 0.28*** | 0.25*** | 0.31*** | 0.26*** |
|  | (0.07) | (0.07) | (0.07) | (0.07) | (0.06) | (0.06) | (0.07) | (0.07) | (0.08) | (0.06) |
| Log Population | 0.35*** | 0.35*** | 0.34*** | 0.36*** | 0.36*** | 0.34*** | 0.34*** | 0.35*** | 0.34*** | 0.34*** |
|  | (0.06) | (0.06) | (0.06) | (0.06) | (0.06) | (0.06) | (0.06) | (0.06) | (0.06) | (0.06) |
| Log Qualitative Articles | 0.62*** | 0.60*** | 0.62*** | 0.57*** | 0.58*** | 0.60*** | 0.62*** | 0.59*** | 0.62*** | 0.57*** |
|  | -5.74*** | -5.60*** | -5.51*** | -6.32*** | -5.74*** | -5.30*** | -5.64*** | -5.48*** | -5.89*** | -5.52*** |
| English is Official Language | 0.42*** | 0.46*** | 0.43*** | 0.47*** | 0.44*** | 0.42*** | 0.44*** | 0.44*** | 0.42*** | 0.43*** |
|  | (0.11) | (0.11) | (0.11) | (0.11) | (0.11) | (0.10) | (0.11) | (0.11) | (0.11) | (0.11) |
| Observations | 166 | 166 | 166 | 166 | 166 | 166 | 166 | 166 | 166 | 166 |
| R Sq. | 0.86 | 0.86 | 0.86 | 0.86 | 0.86 | 0.86 | 0.86 | 0.86 | 0.86 | 0.86 |
| Note: GDP and population data are retrieved from the World Bank's World Development Indicators while the data sources are from the SPI. Regressions include all papers using data from 2017-2019 (36,695 total articles).. ***=0.001 level, **=0.01 level, *=0.05 level, +=0.1 level. | | | | | | | | | | |

Table S3.

Longitudinal Relationships between Number of Papers Using Data and Statistical Performance

|  | (1) | (2) | (3) | (4) | (5) | (6) |
| --- | --- | --- | --- | --- | --- | --- |
| Log GDP per capita | 0.58** |  |  | 0.51 | 0.58** | 0.62** |
|  | (0.20) |  |  | (0.33) | (0.22) | (0.22) |
| Log Population |  | 0.18 |  | 1.09 | 0.40 | 0.38 |
|  |  | (0.35) |  | (0.69) | (0.37) | (0.37) |
| SPI Overall Score |  |  | 0.00 | 0.00 |  |  |
|  |  |  | (0.00) | (0.00) |  |  |
| SPI Overall Score  (Extended Series) |  |  |  |  | 0.01+ |  |
|  |  |  |  |  | (0.00) |  |
| SPI Data Sources Score  (Extended Series) |  |  |  |  |  | 0.00+ |
|  |  |  |  |  |  | (0.00) |
| Observations | 1974 | 1974 | 661 | 661 | 1974 | 1974 |
| R^2^ | 0.957 | 0.956 | 0.991 | 0.991 | 0.957 | 0.958 |
| R^2^ Adj. | 0.953 | 0.952 | 0.988 | 0.988 | 0.954 | 0.954 |
| R^2^ Within | 0.025 | 0.001 | 0.001 | 0.013 | 0.036 | 0.039 |
| R^2^ Within Adj. | 0.025 | 0.000 | -0.001 | 0.007 | 0.035 | 0.037 |
| AIC | 1571.9 | 1621.2 | -280.9 | -285.1 | 1554.1 | 1548.5 |
| BIC | 2421.3 | 2470.6 | 492.0 | 496.8 | 2414.6 | 2409.0 |
| RMSE | 0.33 | 0.34 | 0.15 | 0.15 | 0.33 | 0.33 |
| *Note:* Data from the World Bank’s World Development Indicators (WDI) and SPI. Papers include all papers using data years 2004-2019. 137 countries are included that have an extended SPI value, GDP, and population values in the regression. 68,008 articles are included. SPI Extended Series data supplements SPI data with data from Statistical Capacity Indicator (SCI) to extend series back to 2004. All regressions include standard errors clustered at the country level and country and year fixed effects. ***=0.001 level, **=0.01 level, *=0.05 level,+=0.1 level. | | | | | | |
